# Hepatitis B Viral DNA Decline at Loss of HBeAg Is Mainly Explained by Reduced cccDNA Load – Down-Regulated Transcription of PgRNA Has Limited Impact

**DOI:** 10.1371/journal.pone.0036349

**Published:** 2012-07-20

**Authors:** Sebastian Malmström, Simon B. Larsson, Charles Hannoun, Magnus Lindh

**Affiliations:** Department of Infectious Diseases, University of Gothenburg, Gothenburg, Sweden; Yonsei University, Republic of Korea

## Abstract

**Background:**

Quantification of hepatitis B virus (HBV) DNA and surface antigen (HBsAg) serum levels have become increasingly important for the assessment of clinical stage and response to treatment for chronic hepatitis B. Effective immune clearance results in reduction of viremia by 4–5 log units and HBsAg levels by 2 log, but these processes are not well understood. Thus, it is uncertain to what extent mechanisms that inhibit transcription of the pregenomic RNA (pgRNA), an RNA intermediate, contribute to suppression of viremia. Likewise, it is unclear if transcriptional regulation is important for the excessive production of surface antigen (HBsAg) that is a hallmark of HBV infection.

**Methods:**

HBV RNA and cccDNA were quantified in 19 liver biopsies from patients with chronic HBV infection, as well as in transfected Huh7.5 cells and in PLC/PRF/5 cells carrying integrated HBV genome.

**Results:**

Patients negative for HBeAg had 2.15 log lower levels of cccDNA in liver tissue, 4.84 log lower serum levels of HBV DNA and 1.45 log lower serum levels of HBsAg, than HBeAg-positive patients. The pgRNA in liver tissue correlated strongly with cccDNA (R^2^ = 0.87, p<0.0001) and HBV DNA levels in serum (R^2^ = 0.81, p<0.0001), whereas S-RNA correlated strongly with cccDNA (R^2^ = 0.65, p<0.0001) and HBsAg levels (R^2^ = 0.57, p = 0.0003). The S-RNA/pgRNA ratio was higher in HBeAg-negative patients (ratio 40 vs. 3, p = 0.01) and in PLC/PRF/5 cells, and was in transfected Huh7.5 cells not influenced by mutations in the HBV core promoter.

**Conclusion:**

The reduction of viremia that is observed after loss of HBeAg was mainly explained by reduced cccDNA load in the liver, whereas the contribution of down-regulation of pgRNA transcription was relatively small. Enhanced transcription of S-RNA does not explain excessive production of HBsAg.

## Introduction

Chronic infection with hepatitis B virus (HBV) is present in an estimated 360 million individuals worldwide, and is an important cause of liver cirrhosis and liver cancer [1], [2]. HBV DNA levels in blood, which reflect the rate of viral replication, have a strong impact on the risk for cirrhosis and cancer [3]. As a result of effective immune clearance of infection the hepatitis B e antigen (HBeAg) becomes undetectable in the blood and HBV DNA levels in serum often decrease by more than 5 log from levels around 10^9^ copies/mL, but it is not well known how this reduction of viremia is accomplished. Eradication of infected cells and reduced number of covalently closed circular DNA (cccDNA) copies per infected cell [4]–[6] seem to explain only part of the viral decline (corresponding to ≈ 2 log), and thus additional immune mediated actions have to be involved. The cccDNA, a replicative intermediate of the HBV genome localised to the hepatocyte nucleus, is template for 5 RNA transcripts, among them the pregenomic RNA (pgRNA), which is translated into core and polymerase proteins, and also reverse transcribed into minus strand DNA to form relaxed circular (rc) DNA in the viral capsids. Several reports indicate that replication could be down-regulated by mechanisms that reduce levels of pgRNA from cccDNA, effects that could mediate so-called non-cytolytic control of HBV replication [7], [8]. Such mechanisms include deacetylation of cccDNA-binding histones [9] and interaction with transcription factors binding to the basal core promoter (BCP) [10]–[13].

Hepatocytes produce an excess of the surface protein (HBsAg), which appear in the blood as subviral particles (SVP) at extremely high concentrations, greatly exceeding the levels of virions [14]. The function of SVP is not yet established and it is not well known how the very high production of HBsAg is achieved or regulated. Whereas an effective immune response results in loss of HBeAg and a 4–5 log reduction of HBV DNA levels, the effects on HBsAg levels in blood are moderate with a reduction from 10^5^ IU/mL in HBeAg-positive to around 10^3^ IU/mL in HBeAg-negative patients. The observation that both HBsAg in serum and cccDNA in liver decrease by approximately 2 log at loss of HBeAg [6] suggests that cccDNA in liver tissue might be better reflected by serum levels of HBsAg than by serum levels of HBV DNA. The greater reduction of HBV DNA levels in serum has been interpreted as reduced replication productivity [5], and demonstrate the difference in immune mediated suppression of HBV DNA and HBsAg.

We hypothesised that comparative analysis of different HBV transcripts in liver biopsies and cell cultures would be useful for revealing to what extent mechanisms acting of RNA levels may regulate viral replication or production of HBsAg. Differences in the levels of different RNA species have been observed in vitro by Northern blot, often with HBsAg transcripts being more abundant than pgRNA [15]. By this technique transcripts can be distinguished by their size, but a disadvantage is that quantification based upon measurements of band intensity has a limited range and uncertain accuracy. The application of real-time polymerase chain reaction (PCR) has improved the possibilities of quantifying viral RNA [16], although its utility for studying HBV RNA is to some extent limited by the fact that the different transcripts overlap.

In the present study we utilised a set of real-time PCR assays to study HBV in liver biopsies, with focus on how impact on cccDNA load and transcription efficiency may influence viral replication and HBsAg production. In addition, we investigated hepatoma cells transfected with HBV or carrying integrated HBV DNA in order to learn if transcription in these settings differs from what is observed in vivo.

## Methods

### Patients

Frozen liver biopsies and serum samples drawn at the same time point were investigated. The samples were taken from 19 patients, participating in a study that has been previously described [17]. The patients were infected by genotype A (n = 12) or D (n = 7), and were representative for HBeAg-positive (n = 10) and negative (n = 9) stage of infection and a wide range of viremia levels (Table 1).

**Table 1:** Characteristics of 19 patients whose liver biopsies were analysed.

ID	Age (years)	Genotype	HBeAg	HBsAg(log IU/mL)	HBV DNA(log copies/mL)	ALT/ULN	Histology^a^ Infl/Fibr
167	16	D	+	5.13	10.1	1.50	7/1
83	24	D	+	4.72	9.71	1.88	5/1
117	27	D	+	4.50	9.64	1.15	2/0
115	25	D	+	5.21	9.48	1.83	5/1
2	25	D	+	4.85	8.97	1.25	5/1
77	59	A	+	4.08	8.50	1.63	10/4
114	31	D	+	4.58	7.31	0.82	1/0
152	60	A	+	3.38	7.71	7.00	9/4
130	29	D	+	3.20	7.78	2.00	5/3
139	30	A	+	4.56	6.60	0.88	2/1
101	45	A	–	3.35	5.84	0.57	1/0
127	41	A	–	3.30	4.45	1.0	3/1
68	46	A	–	1.14	4.40	0.40	1/0
60	44	A	–	3.37	3.88	0.50	2/1
99	26	A	–	2.66	3.85	0.49	1/1
55	28	A	–	4.36	3.52	0.65	3/1
105	43	A	–	2.03	3.31	0.65	3/1
65	50	A	–	2.94	3.31	0.36	2/1
63	18	A	–	ND	3.01	0.38	2/0

a Knodell scores for inflammation (sum of interphase, lobular and portal inflammation scores) and fibrosis.

ULN, upper limit of normal; ND, no data.

### Plasmid HBV for Transfection

The two plasmids used in transfection experiments contained one copy of the full-length HBV genome. The wild-type (wt) genome originated from an HBeAg-positive patient with genotype A infection. In the mutant genome an AGG → TGA double mutation at position 1762–64 in the BCP region had been introduced into the wt genome by mutagenesis (confirmed with full-genome sequencing). The HBV genomes were excised from the plasmid by restriction enzyme treatment to obtain linear genomes that were transfected to become template for replication after circularisation within transfected cells [18].

### Transfection of HBV into Human Hepatoma (Huh) Cell Line 7.5

Huh7.5 cell (Apath LLC, St. Louis, MO) were cultured in Dulbecco’s modified medium (DMEM high glucose) supplemented with 10% fetal bovine serum, 0.1 mM nonessential amino acids (NEAA), 100 U of penicillin/mL, and 100 µg of streptomycin/mL in 12-well plates. Initially the wells were seeded with 480 000 cells and after 24 h of growth at 37°C in 5% CO_2_ cells were transfected with 0.625 µg of plasmid DNA using Metafectene Easy (Biontex Laboratories, Germany). After 24 h cells were washed 3 times with 1.5 mL PBS and 1 mL of fresh medium was added. The harvest was performed after another 3 days.

### Hepatoma Cells with HBV Integration

Transcripts from a PLC/PRF/5 cell line [19] (with unknown clone identity) was analysed in order to study the relative levels of pgRNA and S-RNA. This cell line was provided by Dr Göran Bondjers, University of Gothenburg. The expression of these transcripts requires integration also of the corresponding promoters, and has to our knowledge not been investigated, although it is well documented that these cells produce HBsAg but not viral particles. Cells (480 000 cells/well) were grown for 3 days at 37°C in 5% CO_2_, in Dulbecco’s modified medium (DMEM high glucose) supplemented with 10% fetal bovine serum, 2 mM L-glutamine, 0.1 mM nonessential amino acids (NEAA), 1.8 mM sodium pyruvate, 200 U of penicillin/mL, and 200 µg of streptomycin/mL. No transfection was performed on these cells.

### Extraction of DNA and RNA from Cells

DNA and RNA were extracted from transfected and non-transfected hepatoma cells in a Magnapure robot (Roche Applied Science, Germany) using the Total NA protocol. Harvested cells were washed in 1 mL PBS and after centrifugation at 5000 rpm for 3 min the pellet was re-suspended in 800 µL RLT lysis buffer (Qiagen Sciences, MD, USA) before extraction. Half the output from cells were subjected to quantification of DNA by real-time PCR, whereas the other half was treated with DNase (Turbo DNA-free kit, Ambion Inc, TX, USA) before analysis with reverse transcriptase (RT) real-time PCR.

### Preparation of Liver Tissue

Portions (approximately 5 mg) of liver biopsies were investigated. The liver biopsies were stored in –70°C until analysed. After homogenization of the liver tissue in a Magnalyser instrument (Roche), extraction was performed in the Magnapure robot according to the manufacturer’s protocol, using the DNA II Tissue kit. Real-time PCR was performed as outlined below.

### HBV DNA Quantification

The level of HBV DNA in cell culture and liver tissue was measured by real-time PCR primers and probes listed in Table 2. Two primer pairs target segments in the S and C regions, and one pair amplify only cccDNA by spanning the gaps present in the plus and minus strands of the relaxed circular form of the genome that is present in viral particles. Although this PCR is designed to amplify cccDNA, in theory it might also amplify integrated HBV DNA including the targeted segment. Such integrations however seem to be rare, as indicated by observations by Laras et al. who validated this principle for cccDNA detection by comparing results obtained with or without pretreatment with plasmid-safe DNase [16]. Thus, in the interpretation we assume that in liver biopsies this PCR quantifies cccDNA present in infected cells, whereas the same analysis on Huh7.5 cells rather measures the amount of HBV DNA (originating from the cloned plasmid) that has circularised after transfection. In the PLC/PRF/5 cell line, cccDNA results most likely reflect HBV DNA integrated into the human genome. This cell line has been previously characterised, and contains several integrated segments of HBV DNA [19].

**Table 2:** Oligonucleotide sequences of primers and probes.

Target	Oligo	Sequence^a^	Nucleotide position^b^
S-RNA	Forward primer	TCCTCCAAYTTGTCCTGGTYATC	350–372
	Reverse primer	AGATGAGGCATAGCAGCAGGAT	432–410
	Probe (AS)	ATGATAAAACGCCGCAGACACATCCARC	400–373
pgRNA	Forward primer	GGTCCCCTAGAAGAAGAACTCCCT	2367–2390
	Reverse primer	CATTGAGATTCCCGAGATTGAGAT	2454–2431
	Probe	TCTCAATCGCCGCGTCGCAGA	2408–2428
cccDNA	Forward primer	CCGTGTGCACTTCGCTTCA	1575–1593
	Reverse primer	GCACAGCTTGGAGGCTTGA	1882–1864
	Probe	CATGGAGACCACCGTGAACGCCC	1607–1629

a Y, T or C; R, A or G; AS, antisense.

b Position in genotype A genome.

Amplification in 25 µL reaction mixtures containing 12.5 µL Universal PCR master mix (Applied Biosystems, CA, USA), 0.2 µM of each primer and probe, and 5 µL of extracted DNA was performed in an ABI 7300 Real Time PCR System (Applied Biosystems). Initial UNG (50°C, 2 min) and denaturation steps (95°C, 10 min) were followed by 45 cycles of amplification (95°C, 15 s; 60°C, 1 min; 72°C, 10 s). Quantification of betaglobin DNA was used to standardize the HBV DNA results in terms of copies (cp) per human cell equivalent (cEq). All measurements were carried out in duplicates.

### HBV RNA Quantification

Quantification of HBV RNA in liver tissue and cells from cell culture was performed after DNase-treatment, using primers and probes listed in Table 2. After an initial RT step (48°C, 30 min), and denaturation (95°C, 10 min), 45 cycles of amplification (95°C, 15 s; 60°C, 1 min; 72°C, 10 s) were performed. 18S RNA was used as reference for normalisation of RNA levels. All measurements were carried out in duplicates. Each sample was run also with a mastermix without reverse transcriptase to ensure that DNA (potentially remaining after DNase digestion) was not amplified.

Primers in the core region amplify both precore and pregenomic RNA transcripts whereas the S primers amplify also the two S-transcripts. To compensate for variation in amplification efficiency between primers targeting the S and core regions, the Ct values for S-RNA were adjusted by subtracting the mean Ct difference (between S and core) that was observed in DNA PCR using the same primers and probes.

### Serological Tests and Quantification of Serum HBV DNA

HBsAg in serum and medium from cell culture was quantified using the Architect assay (Abbott, Abbott Park, IL). Serum HBV DNA was analysed by Cobas Amplicor HBV Monitor (Roche Diagnostic Systems, Branchburg, NJ). The detection range for the test spans from 10^2^ to 10^5^ copies/mL. To extend this range, samples with levels higher than 10^5^ copies/mL were reanalysed after predilution in negative serum.

### Statistics

Differences in levels of HBV RNA or DNA between groups were analysed by Mann-Whitney U test, and correlations by linear regression and Pearson’s correlation analysis. P values below 0.05 were considered significant. Multiple linear regression was performed to analyse impact of cccDNA level and transcription efficiency of pgRNA (independent variables) on serum level of HBV DNA (dependent variable). The Statview software (SAS Institute) was used for statistical analyses.

### Ethics

After written and verbal information about the study, each patient gave verbal consent to participate, as approved by the Regional Ethics Review Board in Gothenburg.

## Results

### cccDNA PCR

**Figure 1:**
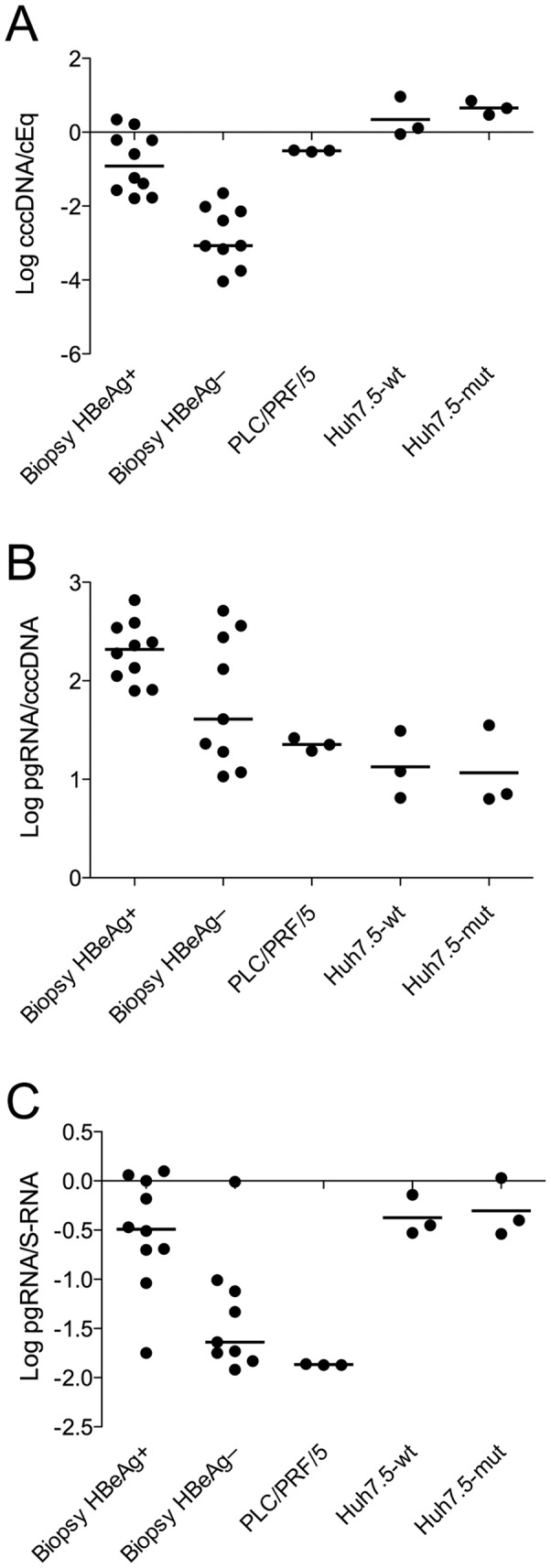
Levels of cccDNA and HBV RNA in vivo and in vitro. The cccDNA levels (A) and pgRNA per cccDNA (B), as well as pgRNA/cccDNA ratios (C) were higher in liver tissue from HBeAg-positive as compared with HBeAg-negative patients. In PLC/PRF/5 cells, the cccDNA PCR amplifies integrated HBV DNA (a segment containing the promoter for pgRNA). In these cells which contain multiple integrations of the S region, the pgRNA/S-RNA ratio was low (C). In Huh7.5 cells, the cccDNA levels, pgRNA per cccDNA and ratio between pgRNA and S-RNA were similar in cells transfected with HBV without or with mutations in the core promoter region, indicating that these mutations have low impact on pgRNA transcription.

The cccDNA content ranged from 0.34 to –4.04 log copies/cell equivalent (cEq) in the 19 liver biopsies (Table 3). The median cccDNA levels were 2.15 log units higher (–0.92 vs. –3.07 log cp/cEq, p<0.0001) in tissue from HBeAg-positive as compared with HBeAg-negative patients (Figure 1A). In Huh7.5 cells, the cccDNA level was 0.11 log copies/cEq in cells transfected with the wt genome, and 0.65 log copies/cEq in cells transfected with the BCP mutant genome (median values in triplicate experiments). The level of DNA amplified by cccDNA PCR in PLC/PRF/5 cells was stable around the median –0.50 log copies/cEq.

**Table 3:** HBV DNA and RNA levels in cell cultures and liver biopsies^a^.

	Liver tissue		PLC/PRF/5 b	Huh7.5 (gt A)^b^		
	HBeAg+ (n = 10)	HBeAg– (n = 9)		wt^c^	mut^c^	*p* d
*Intrahepatic*						
cccDNA/cEq	–0.92 (–1.79–0.34)	–3.07 (–4.04–1.65)	–0.50	0.11	0.65	<0.001
pgRNA/cccDNA	2.32 (1.90–2.82)	1.61 (1.03–2.71)	1.35	1.08	0.85	0.16
S-RNA/cccDNA	2.56 (2.28–3.80)	3.11 (1.29–4.35)	3.21	1.61	1.34	0.24
pgRNA/S-RNA	–0.49 (–1.75–0.10)	–1.64 (–1.92–0.01)	–1.87	–0.45	–0.40	0.01
*Serum*						
HBV DNA (log cp/mL)	8.74 (6.60–10.10)	3.90 (3.00–5.80)	NA	NA	NA	<0.001
HBsAg (log IU/mL)	4.57 (3.20–5.21)	3.12 (1.14–4.36)	NA	NA	NA	0.001

a Median values of log copy numbers, range in parentheses.

b Three repetitive experiments under the same conditions.

c Basal core promoter wild-type (AGG) or mutant (TGA) at 1762–1764.

d Mann-Whitney U test (biopsies).

NA, not applicable.

### HBV RNA Levels

The median levels of pgRNA were higher in biopsies from HBeAg-positive as compared with HBeAg-negative patients (1.69 and –1.20 log copies/cEq, p<0.0001), and the difference was similar for S-RNA. The transcriptional efficiency in terms of pgRNA transcripts per cccDNA (Figure 1B) tended to be lower in HBeAg-negative than in HBeAg-positive biopsies (1.61 vs. 2.32 log cp/cccDNA, p = 0.16). The transcriptional efficiency for pgRNA was also lower in PLC/PRF/5 (1.35 log cp/cccDNA) and in transfected Huh7.5 cells as compared with liver tissue, but there was no difference between BCP wt and mutant genomes (1.08 vs. 0.85 log cp/cccDNA).

The levels of S-RNA were 3 times higher than pgRNA levels (pgRNA/S-RNA ratio –0.49 log) in HBeAg-positive biopsies, and 40 times higher (ratio –1.64 log) in HBeAg-negative biopsies (Figure 1C; p = 0.01). In transfected Huh7.5 cells, the pgRNA/S-RNA ratio was –0.37 log and –0.30 log for BCP wt and mutant respectively. In PLC/PRF/5 cells both transcripts could be detected, at a pgRNA/S-RNA ratio of –1.87 log. HBsAg was detected in medium from Huh7.5 in the range 500–2,200 S/CO and in PLC/PRF/5 in the range 800–1,200 S/CO.

### Correlations

**Figure 2:**
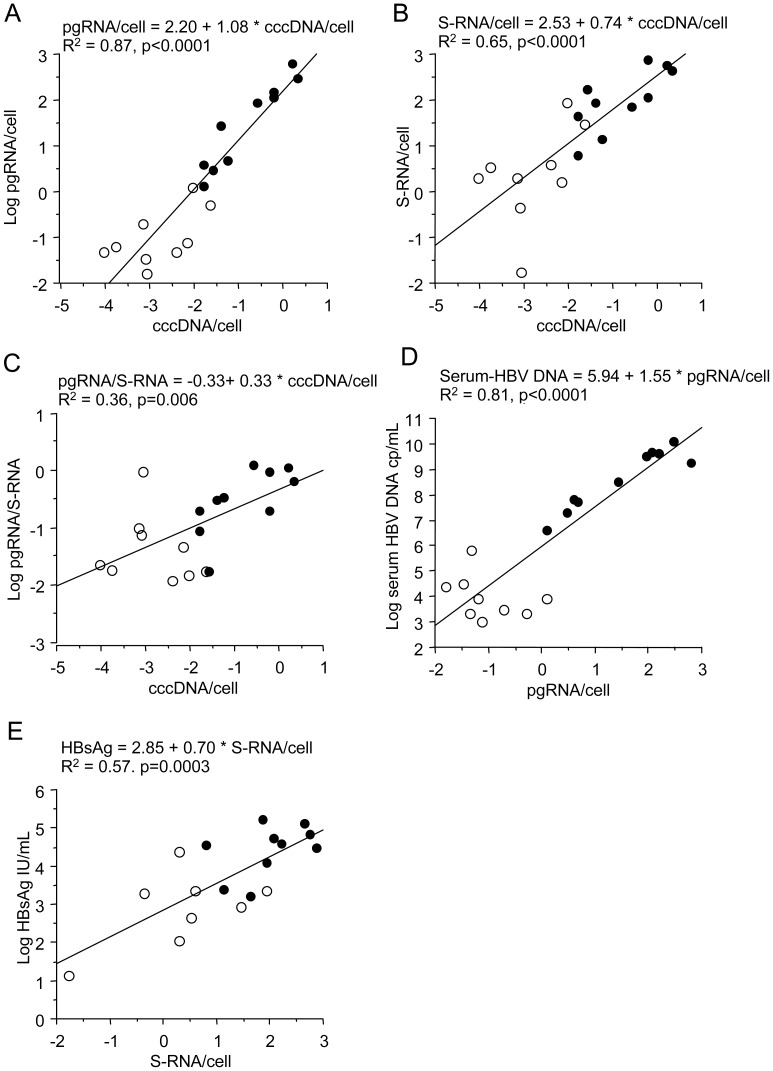
Correlations between different markers for viral productivity in liver and serum. A-C show strong correlation in 19 liver biopsies between cccDNA levels and pgRNA (A) and S-RNA (B), and significant correlation between cccDNA and pgRNA/S-RNA ratio (C). D shows strong correlation between pgRNA and serum levels of HBV DNA (but lack of correlation within the HBeAg-negative subgroup). E shows relatively strong correlation between S-RNA and HBsAg in serum. Filled dots HBeAg positive, open dots HBeAg negative.

The pgRNA in liver tissue correlated strongly with cccDNA (p<0.0001) and with serum levels of HBV DNA (p<0.0001), as shown in Figure 2A and 2D. The correlation between pgRNA and HBV DNA in serum was very strong for all patients and also within the HBeAg-positive group, but there was no correlation within the group of HBeAg-negative patients (Figure 2D). S-RNA levels in liver correlated strongly with cccDNA (p<0.0001) and with serum levels of HBsAg (p = 0.0003), as shown in Figure 2B and 2E.

The high R^2^ value (0.87, Figure 2A) indicates that reduction of cccDNA explains most of the reduction of pgRNA in this set of samples. However, the ratio between pgRNA and S-RNA correlated significantly with cccDNA (p = 0.006, Figure 2C), indicating that pgRNA transcription was suppressed or S-RNA transcription increased in patients with low viremia.

Multiple linear regression analysis showed that in addition to cccDNA (p<0.0001), serum HBV DNA levels were significantly associated with the pgRNA/cccDNA ratio (p = 0.02). The latter finding supports that reduced transcription efficiency of pgRNA may contribute to the reduction of viremia levels.

Markers for liver inflammation (ALT levels and histological inflammation score), but not fibrosis score or age, correlated with cccDNA, pgRNA and serum HBV DNA (R^2^ = 0.42–0.57, p<0.01 for ALT; R^2^ = 0.23–0.33, p<0.04 for inflammation score).

## Discussion

The findings in this study indicate that reduced transcription of pgRNA explain only a relatively small part of the strong suppression of viremia levels that is associated with loss of HBeAg, and that enhanced transcription of S-RNA has limited importance for the very high production of HBsAg that is a hallmark of HBV infection. The patients negative for HBeAg had significantly lower levels of cccDNA (2.15 log), pgRNA (2.89 log) and S-RNA (1.70 log) in liver tissue, lower pgRNA transcription efficiency, and lower levels of HBV DNA (4.84 log) and HBsAg (1.45 log) in serum, as compared with HBeAg-positive patients. Thus, out of the 5 log lower viremia levels observed in HBeAg-negative patients only 2–3 log could be explained by reduced cccDNA viral load and reduced pgRNA transcription.

The pgRNA levels in liver tissue correlated strongly with HBV DNA in serum (p<0.0001, R^2^ = 0.81), but there was also a strong correlation between pgRNA and cccDNA in tissue (p<0.0001, R^2^ = 0.87). The finding that pgRNA levels mainly (>80% as indicated by the R^2^ values) reflect cccDNA viral load suggests that down-regulation of pgRNA transcription efficiency is of limited importance for explaining the suppressed replication, at least the decline associated with loss of HBeAg. However, some of our observations support that regulation of pgRNA may have an impact on replication. The transcriptional efficiency in terms of pgRNA per cccDNA was 0.71 log lower (p = 0.16) in HBeAg-negative patients, and in multiple regression analysis serum levels of HBV DNA were influenced by the pgRNA/cccDNA ratio independently of cccDNA levels in tissue (although less strongly, p = 0.02 vs. p<0.0001). If a reduced transcription was specific for pgRNA, one would expect the ratio between pgRNA and S-RNA in biopsies to decrease after loss of HBeAg, and indeed, the pgRNA/S-RNA ratio was 1.15 log lower in HBeAg-negative patients (p = 0.01). The latter finding may alternatively reflect enhanced transcription of HBsAg, but still these observations suggest that reduced synthesis of pgRNA might contribute to reduced viremia levels, as suggested by observations by others [9], [16], [20]. However, such effects seem to explain a relatively small part of the reduced viral replication that parallels loss of HBeAg.

If immune mediated effects suppress transcription of pgRNA this might promote emergence of mutations in the BCP [21]. In our study, BCP mutations did not, however, influence transcription of pgRNA, but similar pgRNA/cccDNA ratios were observed in Huh7.5 cells transfected with HBV carrying AGG → TGA mutations at nucleotides 1762–1764 as in cells transfected with wild-type virus. This suggests that escape from suppressed replication does not explain the evolution of these mutations, but that the main mechanism for their selection rather would be an impact on HBeAg production [22]. Enhanced replication may still be of importance because in vitro studies have shown that replication increases if further BCP mutations are added [21].

In vitro studies of HBV replication are typically performed by transfection of hepatoma cell lines. This is useful for evaluating for example antiviral resistance, but this approach might not be accurate for evaluating the effects on replication by escape mutations. To assess this we compared transcription efficiencies in transfected cells and liver biopsies, and observed that the pgRNA/cccDNA ratio was approximately 1 log lower in transfected Huh7.5 cells than in the liver tissue. This lower efficiency in vitro as compared with in vivo might reflect that the Huh7.5 cells lack some transcription factors that are present in liver tissue, and suggest that these cells may not be fully representative for the situation in vivo.

The clinical utility of HBsAg quantification has recently gained interest [23]. Several studies have reported that HBsAg levels in serum reflect the cccDNA levels in the liver [24], and others have described that the correlation between serum HBV DNA, HBsAg and cccDNA may vary depending on the stage of the infection [25]. In the present study we found that S-RNA in tissue levels correlated with both cccDNA (p = 0.0001) and serum levels of HBsAg (p = 0.0003). Accordingly, HBsAg levels in HBeAg-negative patients were significantly lower (p = 0.003) than in HBeAg-positive patients, and this difference (1.45 log) was of similar magnitude as the difference in S-RNA (1.70 log), but much smaller than for HBV DNA in serum. Thus, the decline of HBsAg after loss of HBeAg (1.45 log) appears to somewhat underestimate the decline of cccDNA (2.15 log), whereas the decline of HBV DNA (4.84 log) instead is much greater than the reduction of viral load in the liver. From this one cannot conclude which is the best marker for cccDNA, only that they are different, and the observation that cccDNA correlated with HBV DNA with a higher R^2^ than with HBsAg (0.72 vs. 0.54) indicate that HBV DNA levels are less influenced by other factors in untreated patients. Such impact by other factors seem to be of greater importance in HBeAg negative stage, because within HBeAg-negative patients the correlation between cccDNA and both HBV DNA and HBsAg levels in serum was lost, as previously reported by others [25], [26].

Serum HBV DNA levels has become very important as a marker for clinical stage and prognosis, and knowledge about how these levels are regulated may become important for the clinical interpretation. Possibly, analysis of cccDNA in liver biopsies could provide useful information, but in this small study, cccDNA (or pgRNA) did not correlate more strongly with ALT or histological inflammation than did serum HBV DNA. Thus, study of larger number of patients is required to further explore the clinical utility of analysing cccDNA load.

Another aim of the study was to find out if enhanced transcription of S-RNA was important for the very high levels of HBsAg that are produced in almost all patients with HBV infection. Since the concentration in blood of SVP is considered to be ≈ 5 log higher than that of virions (HBV DNA), one would expect the levels of S-RNA to exceed the levels of pgRNA by several log units in magnitude. This was however not the case. The S-RNA level was only ≈ 1 log higher than that of pgRNA, indicating that the very high HBsAg concentration in blood is not a result of highly efficient transcription of S protein genes, but rather may be due to other factors, such as slow degradation of SVP or enhanced translation of S RNA. The ratio between S-RNA and pgRNA was lower in HBeAg-positive than in HBeAg-negative patients, and similar to the ratio observed in transfected Huh7.5 cells. In PLC/PRF/5 cells, which reportedly contain several inserts of the S gene and are known to secrete HBsAg, the ratio was higher and similar to what was observed in HBeAg-negative patients, pointing at the possibility that HBsAg from integrated HBV DNA might contribute to HBsAg in the blood in HBeAg-negative patients with inactive infection.

In summary, the tissue levels of pgRNA and S-RNA closely reflected the levels of cccDNA and correlated strongly with serum levels of HBV DNA and HBsAg. The liver tissue levels of pgRNA were lower in HBeAg-negative patients, indicating that transcription efficiency for pgRNA is reduced in this stage (or alternatively that degradation is faster). However, mechanisms acting on pgRNA appear to explain only a small part of the 5 log lower HBV DNA levels that were seen in HBeAg-negative patients. The excessive production of HBsAg that is characteristic for HBV infection is not due to enhanced transcription of S-RNA.
